# General Audience Engagement With Antismoking Public Health Messages Across Multiple Social Media Sites: Comparative Analysis

**DOI:** 10.2196/24429

**Published:** 2021-02-19

**Authors:** Katja Reuter, Melissa L Wilson, Meghan Moran, NamQuyen Le, Praveen Angyan, Anuja Majmundar, Elsi M Kaiser, Jennifer B Unger

**Affiliations:** 1 Department of Public Health & Preventive Medicine SUNY Upstate Medical University Syracuse, NY United States; 2 Institute for Health Promotion and Disease Prevention Research, Department of Preventive Medicine Keck School of Medicine of USC University of Southern California Los Angeles, CA United States; 3 Division of Disease Prevention, Policy and Global Health, Department of Preventive Medicine Keck School of Medicine of USC University of Southern California Los Angeles, CA United States; 4 Department of Health, Behavior & Society Johns Hopkins Bloomberg School of Public Health Johns Hopkins University Baltimore, MD United States; 5 Southern California Clinical and Translational Science Institute Keck School of Medicine of USC University of Southern California Los Angeles, CA United States; 6 Economic and Health Policy Research American Cancer Society Washington, DC United States; 7 Department of Linguistics, Dornsife College of Letters, Arts and Sciences University of Southern California Los Angeles, CA United States

**Keywords:** affordance, digital, dissemination of science, Facebook, health communication, health promotion, Instagram, online, smoking, social media, tobacco, Twitter, user engagement

## Abstract

**Background:**

Public health organizations have begun to use social media to increase awareness of health harm and positively improve health behavior. Little is known about effective strategies to disseminate health education messages digitally and ultimately achieve optimal audience engagement.

**Objective:**

This study aims to assess the difference in audience engagement with identical antismoking health messages on three social media sites (Twitter, Facebook, and Instagram) and with a referring link to a tobacco prevention website cited in these messages. We hypothesized that health messages might not receive the same user engagement on these media, although these messages were identical and distributed at the same time.

**Methods:**

We measured the effect of health promotion messages on the risk of smoking among users of three social media sites (Twitter, Facebook, and Instagram) and disseminated 1275 health messages between April 19 and July 12, 2017 (85 days). The identical messages were distributed at the same time and as organic (unpaid) and advertised (paid) messages, each including a link to an educational website with more information about the topic. Outcome measures included message engagement (ie, the click-through rate [CTR] of the social media messages) and educational website engagement (ie, the CTR on the educational website [wCTR]). To analyze the data and model relationships, we used mixed effects negative binomial regression, z-statistic, and the Hosmer-Lemeshow goodness-of-fit test.

**Results:**

Comparisons between social media sites showed that CTRs for identical antitobacco health messages differed significantly across social media (*P*<.001 for all). Instagram showed the statistically significant highest overall mean message engagement (CTR=0.0037; 95% CI 0.0032-0.0042), followed by Facebook (CTR=0.0026; 95% CI 0.0022-0.0030) and Twitter (CTR=0.0015; 95% CI 0.0013-0.0017). Facebook showed the highest as well as the lowest CTR for any individual message. However, the message CTR is not indicative of user engagement with the educational website content. Pairwise comparisons of the social media sites differed with respect to the wCTR (*P*<.001 for all). Messages on Twitter showed the lowest CTR, but they resulted in the highest level of website engagement (wCTR=0.6308; 95% CI 0.5640-0.6975), followed by Facebook (wCTR=0.2213; 95% CI 0.1932-0.2495) and Instagram (wCTR=0.0334; 95% CI 0.0230-0.0438). We found a statistically significant higher CTR for organic (unpaid) messages (CTR=0.0074; 95% CI 0.0047-0.0100) compared with paid advertisements (CTR=0.0022; 95% CI 0.0017-0.0027; *P*<.001 and *P*<.001, respectively).

**Conclusions:**

Our study provides evidence-based insights to guide the design of health promotion efforts on social media. Future studies should examine the platform-specific impact of psycholinguistic message variations on user engagement, include newer sites such as Snapchat and TikTok, and study the correlation between web-based behavior and real-world health behavior change. The need is urgent in light of increased health-related marketing and misinformation on social media.

## Introduction

With the emergence of social media, public health organizations face new opportunities and challenges. Social media include widely accessible web-based and mobile information tools that allow users to view, create, and share messages with others on the web [1]. Overall, 72% of American adults and 97% of teens aged 13-17 years teens say they use at least one social media site, many of them daily [2-4]. Public health groups can use social media to instantly reach more people than ever [5-8]. On the other hand, social media users are increasingly exposed to health-related misinformation, polarization, and targeted commercial marketing of potentially health-harming products and practices, and previous work suggests that the public is exposed to widespread antivaccination messages [9,10], e-cigarette endorsements [11-13], and medical misinformation about the COVID-19 pandemic [14]. Messages that promote tobacco, for example, outnumber antitobacco posts on social media, raising concerns about their effects on the users of these sites, especially members of vulnerable populations such as youth [12,15-17]. Public health groups will need to find innovative and cost-effective ways to increase their information output as one way to counterbalance the overabundance of marketing and misinformation.

Public health groups have started to use social media for health promotion to increase awareness of health harms and positively change behavioral intent [18-20]. Evidence-based health promotion and interventions on social media are an area of growing interest among public health groups. A growing number of systematic reviews have examined social media–based interventions for a variety of health topics, diseases, and behavioral risks [21-24]. As suggested by previous research and outlined by social media measurement standards, audience engagement is an important component of social media–based interventions. Engagement goes beyond mere exposure to a social media post and involves the interactions between an audience and an organization and includes activities that indicate acceptance and involvement with a message, such as liking or sharing a post or clicking a link [25-27]. In general, audience engagement with social media messages indicates their interest and involvement and offers possibilities for widespread message dissemination to peers within their networks [28].

Differences in structural layers of communication across social media platforms can play a crucial role in determining the extent of audience engagement with health messages [29]. Different social media platforms have different features that may facilitate audience engagement and, in particular, may facilitate the ability for campaigns to drive audiences to websites or link them with health education programs. For instance, Instagram users may engage with ephemeral content (eg, *stories*) or static content (eg, *posts*). Users may have different privileges regarding their ability to directly link audiences to web content outside of the Instagram platform. Twitter users may subscribe to and engage with content associated with specific hashtags posted by nonnetwork peers, which could facilitate a campaign’s ability to reach a wider audience and connect them to resources offered outside of the social media platform. Facebook users may engage with content posted on private or public community group *pages*, allowing an organization to connect with their audience. Thus, different social media platforms may have different capacity to engage audiences. However, to date, no prior work has systematically examined audience engagement with health promotion messages across social media platforms.

In a health promotion context, addressing the above-mentioned gap is crucial to inform future health promotion communication and intervention strategies [30,31]. Methodologically rigorous studies to investigate the effects of social media as part of health promotion and prevention campaigns are critically needed [24,32] to answer questions such as, Are some social media platforms more effective for public health campaigns than others in terms of getting users’ engagement? Is there a relationship between the number of clicks on health messages and user engagement with the referred to educational website content? Should health groups limit health promotion campaigns to paid advertisements or is it worthwhile investing time in organic (unpaid) social media efforts that rely more heavily on developing and engaging a community of followers? Past social media research has either focused predominantly on general advertising research or on the assessment of health campaigns within the context of a single social media platform [32]. Few studies compared the performance of social media messages across different platforms [33].

This study assesses the difference in user engagement with identical health messages on three social media sites, including Twitter, Facebook, and Instagram. We defined the message effectiveness as digital user engagement, which was assessed via two primary outcome variables: (1) health message engagement: the click-through rate (CTR) of the social media messages and (2) educational website engagement: the CTR of the educational website the messages linked to (wCTR). CTRs are important social media metrics because they indicate the extent to which the audience finds the message appealing or interesting [34] and because many campaigns make social media posts in hopes of driving the audience to educational websites. We hypothesized that the distributed health messages might not get the same engagement on the different social media sites, although these messages are identical and distributed at the same time. More specifically, we aimed to evaluate (1) the effect of the social media platform on user engagement and (2) the impact of the type of message (paid or organic) on user engagement. We chose these three platforms (ie, Twitter, Facebook, and Instagram) because they are among the most popular sites in the United States and are used daily by broad populations across the different age groups [2-4].

Our work contributes to developing a scientific approach for the selection of the appropriate social media platform for a health promotion or intervention and aligns with recent calls for more transparency of the processes and mechanisms that make digital health promotion feasible and effective [35]. If supported, this would provide important knowledge to improve the design of social media–based health education campaigns.

## Methods

### Study Overview

This study included the dissemination and analysis of a total of 1275 antismoking health messages posted across three social media platforms (Twitter, Facebook, and Instagram) between April 19 and July 12, 2017, as previously described [36]. The messages were focused on the risks of using combustible tobacco products. The study target population included English-speaking social media users on Facebook, Twitter, and Instagram.

For this experiment, we developed parameterized text message templates (n=102) and extracted images (n=315) from two government-sponsored health education campaigns [36]. Copy-protected images from these campaigns were replaced with similar images from a public photo repository, Stocksnap, and topic-related hashtags (n=4) from Twitter (eg, #cigs, *#cigarettes,* #smoking, *#tobaccofree)*. All messages were antismoking messages focused on the risk of using combustible tobacco products. However, the messages referred to three different themes: (1) health or appearance or addiction, (2) money, and (3) family, for example, health: *do not let #cigs cut your life short. Smoking #cigarettes can claim more than 10 years of your life;* money: *Smoking half a pack per day costs about $1000/year. Smoking can do serious damage to your wallet;* family: *about 50% of 3- to 11-year-olds are exposed to secondhand smoke. Look out for the lil ones by keeping it #tobaccofree*.

### Theoretical Background

Our hypothesis draws on two theories: the Selective Exposure Self- and Affect-Management (SESAM) model, which posits that selective exposure to media content is driven by pre-existing self-concept, motivations, and affect [37], and the *affordance theory* (also called affordances framework) [38,39], which suggests that social media users’ engagement with health messages depends not only on their needs but also on the characteristics of the social media site. The user interface and features of social media sites influence whether users may or may not perceive or attend to the affordances of a social media site [40-42]. Studies on Facebook, for example, showed that posts requiring a simple user response such as polls might elicit the highest engagement, whereas the most common form of engagement is the use of the *like* feature [43]. This study sheds light on whether there is a relationship between the social media platform and the user engagement with the public health messages distributed on the platform.

### Procedures

Each message was posted at the most once each month for 85 days. To increase the number of message variations, we used related linguistic message variations, for example, using *we* versus *you* versus *they*. As a result, some of the messages appear similar. The messages were randomized and posted once a month. Multimedia Appendix 1 [36] provides the entire list of parameterized message templates used in the experiment. The details of the technology-enhanced implementation of the experiment were previously published, and examples of messages with images for each platform can be found in the technical paper mentioned previously [36].

We used a web-based tool (Trial Promoter) [36] to randomize the order of the messages and disseminated them at the same time in identical form as organic (unpaid) messages and paid advertisements on each social media site. Organic messages are not paid for; they are usually seen by followers and those who are interested in the same topic. In contrast, advertisements are paid messages that can be targeted to broad and hard-to-reach groups of the population based on proprietary information on user demographics and interests owned by the social media site. Paid and organic messages were posted to separate project accounts on each social media site. For the paid messages, we used a set of targeting criteria, such as gender, location, language, and age (as shown in Multimedia Appendix 2).

The daily message volume per social media platform was 6 on Facebook (advertisements and organic), 6 on Twitter (advertisements and organic), and 3 on Instagram (advertisements only because Instagram does not support referral URLs in organic, unpaid messages). On the basis of market research showing that messages sent at these times receive the most user engagement [44,45], we posted messages on Facebook at 9 AM, 1 PM, and 3 PM PST; on Twitter at noon, 3 PM, and 5 PM PST; and on Instagram at 8 AM, 9 AM, and 5 PM PST. The length of the pilot project (85 days) was determined by the available pilot project budget for social media advertisements.

On seeing the message, users could engage with the post by commenting, sharing, liking, and clicking on the link in the message, which directed them to an educational website operational during the campaign period. The website provided more information about the risks of tobacco products, which was based on government-sponsored health education campaigns.

### Data Collection and Confidentiality

Analytics were collected for each distributed message to determine the engagement among social media users with the message and on the referred educational webpage [36]. The information we analyzed for this study is aggregate and nonidentifiable, such as message clicks, message impressions, and website clicks. The data were stored in Trial Promoter [36], which was hosted by the cloud-based hosting provider, Heroku, a Salesforce application. Salesforce has passed security and privacy-related audits and certifications, including the EU-US Privacy Shield Framework and TRUSTe Certification [46]. Study approval was obtained from the institutional review board at the University of Southern California (protocol #HS-16-00660).

### Calculation of Message and Website CTRs

Message effectiveness was defined as digital user engagement, which was assessed via two primary outcome variables: (1) health message engagement: the CTR of the social media messages and (2) educational website engagement: the CTR of the educational website the messages linked to (wCTR). The CTR was defined as the total number of clicks on the message link divided by the total number of impressions for a specific message. Impressions describe the number of times a message was served to potential viewers, as reported by the social media platform. The wCTR was defined as the proportion of those who, after clicking the message link, visited the educational website and scrolled to peruse the content on the landing page.

It was calculated as the number of scrolls on the website divided by the total number of sessions on the website. A session is a group of interactions that a user takes within 30 minutes on a website.

### Data Analysis

#### Differences in Audience Engagement Across Social Media Platforms

We evaluated the message effectiveness across different social media platforms (Facebook, Twitter, and Instagram). The CTR and wCTR were calculated for each social media platform, as described. To evaluate whether the social media platform was associated with the CTR and wCTR, we used mixed effects negative binomial regression with the exposure being the social media type and specifying the message variant as a random effect. We also included the number of impressions or sessions in the model, depending on whether we were estimating the CTR or wCTR, respectively, but constrained the coefficient to 1.0. The models for the wCTR had an overdispersion parameter set to constant=1+delta. A negative binomial Poisson regression model was selected because the data consist of click counts and a large number of messages received 0 clicks, thereby creating an overdispersed distribution.

On the basis of each model, we obtained adjusted predictive margins for the CTR or wCTR by social media site and tested the null hypothesis that there were no differences in the adjusted predictive margins between groups using a z-statistic. No data on user characteristics were collected (ie, data were aggregated by each appearance of the message), and thus, there were no user-specific predictors or confounders available for inclusion in the model. Where data were sparse, we present the results descriptively.

For all models, fit was evaluated via the Hosmer-Lemeshow goodness-of-fit test, plots of residuals, and inspection of model outliers. The Hosmer-Lemeshow test evaluates how well the observed and predicted values from the model align. Both the deviance statistic and Pearson statistic are reported. When the model is inappropriate, the test is statistically significant. Despite multiple comparisons, an unadjusted *P* value of .05 was considered statistically significant, as this study aims to provide preliminary data on this topic. All analyses were conducted using Stata 15.0 (StataCorp).

#### Messages With the Highest Mean Audience Engagement Per Platform

Message ranking is based on mean CTR and wCTR, calculated by the message within each social media platform. The 5 highest ranking messages are reported descriptively for each platform because of the sparseness of the data.

#### Differences in Audience Engagement With Organic Messages and Paid Advertisements

We also examined the effect of organic, unpaid messages versus paid advertisements on the CTR, independent of the social media platform. The data were too sparse to examine the independent effects on wCTR, so we present unadjusted values. As described previously, we used multilevel mixed effects negative binomial regression to model the relationship between the CTR and wCTR and the type of message (organic message vs paid advertisement). In the CTR model only, we included the covariate social media type. Predictive margins were calculated, and differences were tested between groups as described earlier.

### Statistical Power

Statistical power was calculated to evaluate the message effectiveness via evaluation of the CTR across the selected social media platforms (Twitter, Facebook, and Instagram). We calculated the sample size using a mixed effects negative binomial model. Data from a web-based smoking cessation study through Facebook (FB) suggested a CTR^FB^ of 0.18% [47]. A publication reporting data from an academic health care Twitter (T) account noted a CTR^T^ of 2.01% [48]. Owing to the wide range of reported CTRs and the paucity of the available data, we considered it prudent to use a slightly more conservative estimate for CTR^T^ of 1.50%. There is currently no reported literature providing data from which to calculate the CTR^Instagram^ for Instagram. On the basis of the estimates provided, as well as assuming 80% power, an α level of .05, an exposure time of 1.0, and a negative binomial dispersion of 1.0, we obtained an estimate of 1176 messages required. One of the goals of this study is to estimate the effect size for future, well-powered studies. Power was calculated using PASS software, version 14 (NCSS, LLC).

### Exclusion of Data

Paid messages required approval on all three platforms. The number of days we ran each advertisement was limited to 3 days. However, some paid messages received zero impressions because of the delay in approval of up to 3 days. We excluded these missing values generated for the CTR (n=148) and wCTR (n=524) because of impressions or sessions (the denominator) equaling 0, making it impossible to calculate CTR or wCTR, respectively. To evaluate the influence of the message theme, we further excluded observations (CTR: n=29; wCTR: n=21), where the message theme (ie, health, appearance, money, or family) was unclear. Finally, we excluded observations for the CTR, where the number of impressions was completely missing (n=6). The remaining number of messages for analysis was 1062 for the CTR and 700 for the wCTR.

## Results

### CTR by Social Media Platform

This health communication experiment included a total of 1275 antismoking health messages that were distributed across three social media sites: Twitter, Facebook, and Instagram. All comparisons between the types of social media used in this experiment showed CTRs that differed significantly from one social media platform to the other (*P*<.001 for all; Table 1). More specifically, the CTR for Instagram was the highest, followed by Facebook and Twitter (Multimedia Appendix 3).

**Table 1:** Comparisons of CTRs for the analysis of 1275 antismoking health messages that were posted across three social media platforms (Twitter, Facebook, and Instagram) between April 19 and July 12, 2017.

Social media type	Total clicks	Total impressions	CTR^a^ (95% CI)	*P* values^b^ for the comparisons of the CTRs among the three social media types		
				Facebook	Instagram	Twitter
Facebook	510	504	0.0026 (0.0022-0.0030)	N/A^c^	<.001	<.001
Instagram	255	251	0.0037 (0.0032-0.0042)	N/A	N/A	<.001
Twitter	510	484	0.0015 (0.0013-0.0017)	N/A	N/A	N/A

^a^CTR: click-through rate.

^b^*P* values were obtained using multilevel mixed effects negative binomial regression.

^c^N/A: not applicable.

### Website CTR by Social Media Type

Pairwise comparisons of the social media types differed with respect to the wCTR (*P*<.001 for all; Table 2). The wCTR for Twitter was the highest, followed by Facebook and Instagram (Multimedia Appendix 3).

**Table 2:** Comparison of website CTR by social media type for analysis of 1275 antismoking health messages that were posted across three social media platforms (Twitter, Facebook, and Instagram) between April 19 and July 12, 2017.

Social media type	CTR^a^ (95% CI)	*P* values^b^ for the comparisons of the CTRs among the three social media types		
		Facebook	Instagram	Twitter
Facebook	0.2213 (0.1932-0.2495)	N/A^c^	<.001	<.001
Instagram	0.0334 (0.0230-0.0438)	N/A	N/A	<.001
Twitter	0.6308 (0.5640-0.6975)	N/A	N/A	N/A

^a^CTR: click-through rate.

^b^*P* values were obtained using multilevel mixed effects negative binomial regression.

^c^N/A: not applicable.

### Independent Effects of Paid Advertisements Versus Organic Messages

After adjusting for social media type, we found that the type of post, organic (unpaid) or paid, was statistically significantly associated with the CTR (Table 3). Specifically, organic messages had a higher CTR than paid advertisements. In addition, the adjusted CTRs for social media types were also significantly different from one another (*P*<.001 for all), with Instagram having the highest CTR, followed by Facebook and Twitter.

We also found that the type of post, organic (unpaid), or paid, was statistically significantly associated with wCTR (Multimedia Appendix 4). However, in contrast to the message engagement (CTR), organic messages had a lower wCTR compared with paid advertisements (*P*<.001). We were not able to adjust the wCTRs for social media types because the data became sparse in some categories, and the statistical model would not converge.

**Table 3:** Effects of message type on click-through rate adjusted for social media type for the analysis of 1275 antismoking health messages that were posted across three social media platforms (Twitter, Facebook, and Instagram) between April 19 and July 12, 2017.

Variable		n (%)	Click-through rate^a^ (95% CI; n=1062)	Comparison	*P* value^b^
**Advertisement type**					
	Organic	346 (27.1)	0.0074 (0.0047-0.0100)	Organic versus paid	<.001
	Paid	716 (56.2)	0.0022 (0.0017-0.0027)	Organic versus paid	<.001
**Social media type**					
	Facebook	346 (27.1)	0.0043 (0.0030-0.0057)	Instagram versus Facebook	<.001
	Instagram	245 (19.2)	0.0064 (0.0045-0.0084)	Instagram versus Twitter	<.001
	Twitter	471 (36.9)	0.0022 (0.0016-0.0028)	Twitter versus Facebook	<.001

^a^All estimates are mutually adjusted.

^b^*P* values were obtained using multilevel mixed effects negative binomial regression, followed by the calculation of marginal means.

### Effects of Message and Image Themes on CTR and wCTR

All messages were antismoking messages. However, they referred to three different themes: (1) health or health and community or health and family, (2) money, and (3) addiction. We did not find an effect of the message theme (love of family [LOF] vs no LOF) on the CTR or wCTR (CTRLOF=0.0024, 95% CI 0.0019-0.0029 versus CTRnoLOF=0.0027, 95% CI 0.0023-0.0030; *P*=.33) or wCTR (wCTRLOF=0.1451, 95% CI 0.1039-0.1863 vs wCTRnoLOF=0.1746, 95% CI 0.1549-0.1942; *P*=.20). We further investigated whether an interaction existed between social media type and image theme for both CTR and wCTR but found no statistically significant interaction with either CTR (*P*=.48) or wCTR (*P*=.21).

### Messages With Highest CTR

Of the 1275 distributed messages, the message with the highest CTR was “Smoking can destroy the tiny hairs that help keep the lungs clear, giving a person a smoker’s cough” on Facebook. However, Facebook also had some of the lowest CTRs, resulting in a lower mean overall CTR (Table 4). Only the health message “Polonium-210 is a chemical in #cigarette smoke. It’s also found in nuclear reactors” was found to have a high mean CTR on more than one platform.

**Table 4:** Top 5 messages by social media platform that showed the highest click-through rates for analysis of 1275 antismoking health messages that were posted across three social media platforms (Twitter, Facebook, and Instagram) between April 19 and July 12, 2017.

Platform and health message			Theme		Mean click-through rate		Number of clicks		Number of impressions^a^
**Facebook**									
	Smoking can destroy the tiny hairs that help keep the lungs clear, giving a person a smoker’s cough.	Health		0.201		2		1919	
	How does #smoking take a decade of life away? Smokers die about 12% earlier than nonsmokers.	Health		0.007		10		1595	
	Nicotine can change the way a person's brain works, causing them to crave more and more nicotine.	Addiction		0.006		8		1599	
	Smoking half a pack per day costs about $1000/year. Smoking can do serious damage to your wallet.	Money		0.006		12		1942	
	Tobacco use causes 1300 US deaths daily-more than AIDS, alcohol, car accidents, homicides & illegal drugs combined.	Health		0.006		5		934	
**Instagram**									
	Don’t let #cigs cut your life short. Smoking #cigarettes can claim more than 10 years of your life.	Health		0.023		15		622	
	About 20% of all US deaths are caused by a #smoking-related disease. Forget death, chase life.	Health		0.020		14		707	
	Smoking can cause cancer almost anywhere in the body. 160,000+ US cancer deaths every year are linked to #smoking.	Health		0.014		19		3573	
	Smoking #cigarettes can claim more than 10 years of your life. Don’t let #cigs cut your life short.	Health		0.014		19		2869	
	Polonium-210 is a chemical in #cigarette smoke. It's also found in nuclear reactors.	Health		0.013		12		1292	
**Twitter**									
	480,000 US deaths are caused by a #smoking-related disease every year. Forget death, chase life.	Health		0.035		10		5121	
	Polonium-210 is a chemical in #cigarette smoke. It's also found in nuclear reactors.	Health		0.032		9		851	
	About 40% of nonsmokers in this country are exposed to toxic secondhand smoke.	Health		0.014		12		7434	
	Over 100 million nonsmokers in this country are exposed to toxic secondhand smoke.	Health		0.014		6		4658	
	Over 160,000 cancer deaths in the US every year are linked to #smoking.	Health		0.014		9		10000	

^a^Impressions: number of times a post or advertisement is displayed, whether or not the post is clicked.

Overall, Twitter had the highest wCTRs, followed by Facebook (Table 5). A variety of themes were represented in the messages that received website clicks and were thus examined by the wCTR, although the predominant themes remained to be health-related, including appearance and addiction (12/15, 80%) as opposed to money or family. One health message had a high mean wCTR on both Twitter and Facebook, “Smokers die about 10 years younger than nonsmokers. When someone dies from #tobacco use, we lose them too soon,” suggesting potential resonance with a cross-platform population.

**Table 5:** Top 5 messages by social media type that showed the highest website click-through rate for the analysis of 1275 antismoking health messages that were posted across three social media platforms (Twitter, Facebook, and Instagram) between April 19 and July 12, 2017.

Platform and health message			Theme		Mean website click-through rate		Number of clicks		Number of sessions^a^
**Facebook**									
	Smoking can shorten your life by 10 years. If you smoke, you may be cutting your time with the fam short	Health and family		0.750		4		7	
	Smokers die about 10 years younger than nonsmokers. When someone dies from #tobacco use, we lose them too soon	Health and community		0.578		8		10	
	On average, every cig reduces your life by 11 minutes. Even occasional #smoking can hurt you	Health		0.515		15		17	
	#Smoking can damage our wallets. Smoking half a pack per day costs $1000 per year on average	Money		0.512		10		15	
	Polonium-210 is a chemical in nuclear reactors. It’s also found in #cigarette smoke	Health		0.500		2		4	
**Instagram**									
	How does #smoking take a decade of life away? Smokers die about 12% earlier than nonsmokers	Health		0.500		1		6	
	In the US, 480,000 deaths are caused by a #smoking-related disease every year	Health		0.383		9		13	
	Nicotine can change the way your brain works, causing you to crave more and more nicotine	Addiction		0.357		13		14	
	Over 100 million nonsmokers in this country are exposed to toxic secondhand smoke	Health		0.333		14		14	
	#Smoking can weaken the immune system, leaving a person more vulnerable to bronchitis & pneumonia	Health		0.200		8		17	
**Twitter**									
	There is no safe level of exposure to secondhand smoke. Even a short time can harm people’s health	Health		1.0		4		2	
	There is no safe level of exposure to secondhand smoke. Even a short time can harm our health	Health		1.0		7		2	
	Teens underestimate how addictive #cigarettes are. 3 out of 4 teen smokers become adult smokers	Addiction		1.0		7		2	
	Smoking can cause cancer almost anywhere in the body. 160,000+ US cancer deaths every year are linked to #smoking	Health		1.0		6		2	
	Smokers die about 10 years younger than nonsmokers. When someone dies from #tobacco use, we lose them too soon	Health and community		1.0		4		2	

^a^Sessions: a session is defined as a group of interactions that a user takes within a time frame of 30 minutes on a website.

## Discussion

### Principal Findings

This study tested 1275 antitobacco public health messages, targeting English-speaking users in the United States on three popular social media platforms (Twitter, Facebook, and Instagram). The results demonstrate that the same public health message received different levels of engagement from social media users, depending on the platform. Instagram, a platform that focuses on helping users to share photos and video content, showed the statistically significant highest overall mean CTR compared with Facebook and Twitter. However, for any individual message, Facebook resulted in the highest and lowest CTRs, indicating that Facebook might generate the highest level of user engagement for an individual message while also posing the highest risk of underperforming messages.

We also assessed whether users visited the educational website and engaged with its content (ie, scrolling down to peruse the content on the landing page). The findings show that engagement with a health message on social media (ie, measured as CTR in this study) does not indicate user engagement on the website. Health messages on Twitter showed low CTRs, but they resulted in the highest level of website engagement (wCTR), followed by Facebook and Instagram. Therefore, it is recommended that both metrics (CTR and wCTR) should be taken into account when designing health promotion strategies.

The difference in user engagement, which we found for paid, advertised versus organic messages, was surprising. Paid advertisements on social media allow the targeting of special group characteristics, such as age, gender, language, interests, and location. At the same time, organic messages rely more heavily on developing and engaging a community of followers. Our data suggest that unpaid, organic messages deserve equal engagement when designing health interventions. In this study, organic messages showed significantly higher CTRs than paid advertisements, suggesting the importance of combining organic and advertising messages in health promotion campaigns. Users might distrust advertisements as messages that are designed to manipulate them into buying something. However, we believe this is less likely, as the opposite seems to be the trend. Marketers and publishers are increasingly using forms of *native advertising*, that is, content that bears a similarity to the news, feature articles, product reviews, entertainment, and other materials that surround it on the web. In 2015, before this study was conducted, the Federal Trade Commission Act prohibited deceptive or unfair practices. It issued an Enforcement Policy Statement on Deceptively Formatted Advertisements that explains how the agency applies established truth-in-advertising standards in this context [49]. At the same time, the increased emphasis on advertising transparency on social media platforms was initiated after this study was conducted. Twitter, for example, updated its Political Content Policy on November 22, 2019 [50]. Future research needs to examine the extent to which this affects the advertising of public health messages.

### Application of Theory

We kept the message content, including the image and distribution modus (ie, date of a message, time of day distributed) consistent across the three social media sites. Hence, we discuss the results under the assumption that users’ probability of being exposed to the health messages did not differ across social media sites. This allows us to discuss our findings in light of potential platform-specific factors that may have contributed to the difference in user engagement with the messages: first, user factors that may affect their selected exposure to content, and second, technical site features.

The SESAM model posits that user motivations for selectively exposing themselves to media content vary situationally [37]. This is supported by research showing that users’ motivation to use a specific social media site may vary, which may, in turn, be associated with different levels of engagement with health promotion messages. The Pew Research Center, for example, reported that Twitter is one of the social media sites with the most news-focused users [51]. According to the Pew Research Centre, “around seven-in-ten American adult Twitter users (71%) get news on the site” [51]. Thus, Twitter users may be more focused on their use of the platform and click more exclusively on content that serves this particular motivation. This could explain why, despite a lower CTR, the Twitter messages in this study resulted in the highest website engagement level. However, Facebook is a site where more than half of the users are exposed to news-related content (67%) [52]. These findings indicate that news-like posts may perform better on these platforms. Future research could test the hypothesis of whether health messages that apply characteristics of news are more effective in generating message engagement on these platforms.

On the other hand, Instagram has less of a news focus among its user base [52]. Users’ primary motives for using the site have been described, for example, surveillance and knowledge about others, documentation, coolness, creativity, and escapism [53,54]. Thus, users may have clicked on the content we posted for these motivations (ie, curiosity or to find out what the content was about), but not been motivated to click further through to the website because the initial motivation (surveillance) was satisfied by the initial click. The different kinds of user intent could explain the higher CTR but lower engagement with informative content. Our data suggest that the nature of intent may play a role in engaging with subsequent educational content promoted. Health promotion messages on Instagram could be less focused on referring users to a website. Instead, they deliver instant access to the information on Instagram itself. If future research demonstrates the effectiveness of this approach, organizations could consider using Instagram posts that immediately convey the relevant information to users, rather than using posts that require users to connect through to a website to obtain the relevant information. In addition, organizations could consider posting Instagram content that is visually compelling. All three social media sites allow users to include visual media (ie, images, videos) in their posts, but Instagram stands out as a platform that emphasizes visual content over text [55]. The emphasis on visual content on Instagram might have affected the users’ propensity to engage with the health messages. Research has shown that digital forms of media monopolize individuals’ engagement and attention spans, using visual strategies that demand our interactions [56].

Second, specific platform features might have contributed to the difference in user message engagement and behavior. Bucher and Helmond [39] introduced the *concept of affordance* of social media platforms to understand and analyze social media interfaces and the relations between technology and its users. They suggested that social media users may or may not *perceive or attend to the affordance* of a social media site according to their needs but also to the affordances (ie, technical features) of the social media site—in this case, engage with the message and click on its link. However, the technical features of the three social media sites used in this study are similar, allowing users to interact with content through *likes*, shares (retweets), and comments (replies) to a message. The success of applications such as Facebook relies on the simplicity and immersive design of their interface. Internet platforms are designed to capture viewers’ engagement [56]. We argue that this applies to the three platforms used here. Nonetheless, there may still be nuances in site design that facilitate clicks, for example, Instagram’s focus on visual imagery [57] may contribute to a higher CTR.

### Study Limitations

This pilot study has multiple limitations. Owing to the limited advertisement budget, we had to limit the study to 85 days, which limited the amount of data we could gather and analyze. The analysis is further limited to the digital data that we could access, not including potential behavior change and other real-world implications. The data itself are in aggregate, which do not allow the analysis of demographic or behavioral factors that might have influenced the outcomes assessed here. To increase the number of message variations, we used linguistic variations, for example, using *we* versus *you* versus *they*. However, for some variables, data were too sparse to adjust for all covariates of interest. The study was also underpowered to detect significant interactions of interest.

Furthermore, the generalizability of this study is limited. The messages we tested were antitobacco messages with nuanced messaging focused on either the health effect of smoking, the cost of tobacco products, or the negative impact on family members such as siblings. Health education campaigns targeting other health behaviors might show different results.

Finally, in this study, it is difficult to discern the effect of the message text from the message image’s impact on the CTR and wCTR. Although our data do not suggest that the image theme influenced the CTR or wCTR, other research has demonstrated imagery as a strong predictor of user engagement [58]. Rus and Cameron [58] showed that messages with images had higher rates of liking and sharing relative to messages without images on Facebook. The formats and demographics of social media sites are constantly changing and have evolved since the study was conducted.

### Conclusions

Our study provides evidence-based insights to guide the design of health promotion efforts on social media. Using the full potential of social media for health promotion efforts will require a deeper understanding of the factors that drive user message engagement across different social media to ultimately support informed health decisions and positive behavior change.

Future studies should examine the platform-specific impact of psycholinguistic message variations on user engagement and include newer sites such as Snapchat and TikTok. We suggest a focus on more rigorous studies and a move toward *evidence-based science communication* [59] and study the correlation between web-based behavior and real-world health behavior change. The need is urgent in light of increased health-related marketing and misinformation on social media, as evidenced most recently through the COVID-19 infodemic [60], a global epidemic of misinformation spreading rapidly through social media and other outlets that poses a serious problem for public health.

## Appendix

Multimedia Appendix 1 List of parameterized message templates used in the experiment. Examples of messages with images for each platform can be found in the technical implementation paper of the experiment mentioned above.

Multimedia Appendix 2 Targeting criteria for paid messages (advertisements) on Twitter, Facebook, and Instagram.

Multimedia Appendix 3 Message impressions, message clicks, website sessions, and website clicks per message.

Multimedia Appendix 4 Effects of message type (paid advertisements vs organic messages) on website click-through rate for the analysis of 1275 antismoking health messages that were posted across 3 social media platforms (Twitter, Facebook, and Instagram) between April 19 and July 12, 2017.

## Abbreviations

CTR: click-through rate
FB: Facebook
FDA: Food and Drug Administration
IG: Instagram
NIH: National Institutes of Health
SESAM: Selective Exposure Self- and Affect-Management
T: Twitter
wCTR: website click-through rate
