# Correction: General Audience Engagement With Antismoking Public Health Messages Across Multiple Social Media Sites: Comparative Analysis

**DOI:** 10.2196/28131

**Published:** 2021-02-24

**Authors:** Katja Reuter, Melissa L Wilson, Meghan Moran, NamQuyen Le, Praveen Angyan, Anuja Majmundar, Elsi M Kaiser, Jennifer B Unger

**Affiliations:** 1 Department of Public Health & Preventive Medicine SUNY Upstate Medical University Syracuse, NY United States; 2 Institute for Health Promotion and Disease Prevention Research, Department of Preventive Medicine Keck School of Medicine of USC University of Southern California Los Angeles, CA United States; 3 Division of Disease Prevention, Policy and Global Health, Department of Preventive Medicine Keck School of Medicine of USC University of Southern California Los Angeles, CA United States; 4 Department of Health, Behavior & Society Johns Hopkins Bloomberg School of Public Health Johns Hopkins University Baltimore, MD United States; 5 Southern California Clinical and Translational Science Institute Keck School of Medicine of USC University of Southern California Los Angeles, CA United States; 6 Economic and Health Policy Research American Cancer Society Washington, DC United States; 7 Department of Linguistics, Dornsife College of Letters, Arts and Sciences University of Southern California Los Angeles, CA United States

In “General Audience Engagement With Antismoking Public Health Messages Across Multiple Social Media Sites: Comparative Analysis” (JMIR Public Health Surveill 2021;7(2):e24429) the authors noted errors in the affiliations of two authors.

For authors Katja Reuter and Jennifer B Unger, the affiliation:

Institute for Health Promotion and Disease Prevention Research, Department of Preventive Medicine, Keck School of Medicine of USC, University of Southern California, Los Angeles, California, United States

was inadvertently removed and replaced with the affiliation:

Economic and Health Policy Research, American Cancer Society, Washington, DC, United States

In the originally published paper, the full list of authors and affiliations read as follows:

Katja Reuter^1,2*^, PhD; Melissa L Wilson^3*^, PhD; Meghan Moran^4^, PhD; NamQuyen Le^5^, MPH; Praveen Angyan^5^, MS; Anuja Majmundar^2^, MA, MBA, PhD; Elsi M Kaiser^6^, MS, PhD; Jennifer B Unger^2*^, PhD

^1^Department of Public Health & Preventive Medicine, SUNY Upstate Medical University, Syracuse, NY, United States

^2^Economic and Health Policy Research, American Cancer Society, Washington, DC, United States

^3^Division of Disease Prevention, Policy and Global Health, Department of Preventive Medicine, Keck School of Medicine of USC, University of Southern California, Los Angeles, CA, United States

^4^Department of Health, Behavior & Society, Johns Hopkins Bloomberg School of Public Health, Johns Hopkins University, Baltimore, MD, United States

^5^Southern California Clinical and Translational Science Institute, Keck School of Medicine of USC, University of Southern California, Los Angeles, CA, United States

^6^Department of Linguistics, Dornsife College of Letters, Arts and Sciences, University of Southern California, Los Angeles, CA, United States

^*^these authors contributed equally

The corrected list of authors and affiliations is as follows:

Katja Reuter^1,2*^, PhD; Melissa L Wilson^3*^, PhD; Meghan Moran^4^, PhD; NamQuyen Le^5^, MPH; Praveen Angyan^5^, MS; Anuja Majmundar^6^, MA, MBA, PhD; Elsi M Kaiser^7^, MS, PhD; Jennifer B Unger^2*^, PhD

^1^Department of Public Health & Preventive Medicine, SUNY Upstate Medical University, Syracuse, NY, United States

^2^Institute for Health Promotion and Disease Prevention Research, Department of Preventive Medicine, Keck School of Medicine of USC, University of Southern California, Los Angeles, California, United States

^3^Division of Disease Prevention, Policy and Global Health, Department of Preventive Medicine, Keck School of Medicine of USC, University of Southern California, Los Angeles, CA, United States

^4^Department of Health, Behavior & Society, Johns Hopkins Bloomberg School of Public Health, Johns Hopkins University, Baltimore, MD, United States

^5^Southern California Clinical and Translational Science Institute, Keck School of Medicine of USC, University of Southern California, Los Angeles, CA, United States

^6^Economic and Health Policy Research, American Cancer Society, Washington, DC, United States

^7^Department of Linguistics, Dornsife College of Letters, Arts and Sciences, University of Southern California, Los Angeles, CA, United States

^*^these authors contributed equally

The correction will appear in the online version of the paper on the JMIR Publications website on February 24, 2021, together with the publication of this correction notice. Because this was made after submission to PubMed, PubMed Central, and other full-text repositories, the corrected article has also been resubmitted to those repositories.

