# Supplementary material for: General Audience Engagement With Antismoking Public Health Messages Across Multiple Social Media Sites: Comparative Analysis
Source: JMIR Public Health Surveill. 2021 Feb 19;7(2):e24429. doi: 10.2196/24429 (PMC7935649; doi:10.2196/24429)
Supplement: Multimedia Appendix 1 [file publichealth_v7i2e24429_app1.docx]

**Multimedia Appendix 1. List of parameterized message templates used in the experiment.** Hashtags are only assigned to those messages that don’t already include any hashtags.

| **Message content** | **Hashtags** | **Theme** |
| --- | --- | --- |
| #Smoking damages your DNA, which can lead to cancer almost anywhere in your body. |  | health |
| #Smoking damages our DNA, which can lead to cancer almost anywhere in our bodies. |  | health |
| #Smoking damages DNA, which can lead to cancer almost anywhere in the body. |  | health |
| Even if you don’t smoke, you could be breathing in the deadly mix by being around smokers. | #smoking, #tobacco, #cigs, #cigarettes | health |
| Even if we don’t smoke, we could be breathing in the deadly mix by being around smokers. | #smoking, #tobacco, #cigs, #cigarettes | health |
| Even if someone doesn't smoke, they could be breathing in the deadly mix by being around smokers. | #smoking, #tobacco, #cigs, #cigarettes | health |
| There is no safe level of exposure to secondhand smoke. Even a short time can harm your health. | #smoking, #tobacco, #cigs, #cigarettes | health |
| There is no safe level of exposure to secondhand smoke. Even a short time can harm our health. | #smoking, #tobacco, #cigs, #cigarettes | health |
| There is no safe level of exposure to secondhand smoke. Even a short time can harm people's health. | #smoking, #tobacco, #cigs, #cigarettes | health |
| Using #tobacco products can lead to addiction and expose you to toxic, cancer-causing chemicals. |  | health |
| Using #tobacco products can lead to addiction and expose us to toxic, cancer-causing chemicals. |  | health |
| Using #tobacco products can lead to addiction and expose people to toxic, cancer-causing chemicals. |  | health |
| Some say they smoke to reduce stress. Nope. #Smoking can make your feelings of stress even worse. |  | unclear |
| Some say they smoke to reduce stress. Nope. #Smoking can make our feelings of stress even worse. |  | unclear |
| Some say they smoke to reduce stress. Nope. #Smoking can make feelings of stress even worse. |  | unclear |
| Nicotine can change the way your brain works, causing you to crave more and more nicotine. | #smoking, #tobacco, #cigs, #cigarettes | addiction |
| Nicotine can change the way our brains work, causing us to crave more and more nicotine. | #smoking, #tobacco, #cigs, #cigarettes | addiction |
| Nicotine can change the way a person's brain works, causing them to crave more and more nicotine. | #smoking, #tobacco, #cigs, #cigarettes | addiction |
| #Smoking can damage your wallet. Smoking half a pack/day costs $1000 per year on average. |  | money |
| #Smoking can damage our wallets. Smoking half a pack/day costs $1000 per year on average. |  | money |
| #Smoking can damage everyone's wallets. Smoking half a pack/day costs $1000 per year on average. |  | money |
| #Smoking can weaken your immune system, leaving you more vulnerable to bronchitis & pneumonia. |  | health |
| #Smoking can weaken our immune systems, leaving us more vulnerable to bronchitis & pneumonia. |  | health |
| #Smoking can weaken the immune system, leaving a person more vulnerable to bronchitis & pneumonia. |  | health |
| #Smoking damages your DNA, which can cause cancer almost anywhere, not just your lungs. |  | health |
| #Smoking damages our DNA, which can cause cancer almost anywhere, not just our lungs. |  | health |
| #Smoking damages DNA, which can cause cancer almost anywhere, not just the lungs. |  | health |
| #Smoking can destroy the tiny hairs that help keep your lungs clear, giving you smoker’s cough. |  | health |
| #Smoking can destroy the tiny hairs that help keep our lungs clear, giving us smoker’s cough. |  | health |
| #Smoking can destroy the tiny hairs that help keep the lungs clear, giving a person smoker’s cough. |  | health |
| Smoking #cigarettes can claim more than 10 years of your life. Don’t let #cigs cut your life short. |  | health |
| Don’t let #cigs cut your life short. Smoking #cigarettes can claim more than 10 years of your life. |  | health |
| 3 out of 4 teen smokers become adult smokers. Teens underestimate how addictive #cigarettes are. |  | addiction |
| Teens underestimate how addictive #cigarettes are. 3 out of 4 teen smokers become adult smokers. |  | addiction |
| Every day in the US 700+ youth become daily smokers. Nicotine is highly addictive. | #smoking, #tobacco, #cigs, #cigarettes | addiction |
| Nicotine is highly addictive. Every day in the US 700+ youth become daily smokers. | #smoking, #tobacco, #cigs, #cigarettes | addiction |
| Over 16 million Americans have a disease caused by #smoking. Smoking can cause long-term damage. |  | health |
| #Smoking can cause long-term damage. Over 16 million Americans have a disease caused by smoking. |  | health |
| Secondhand smoke causes 40,000+ US deaths per year. There is no safe level of exposure. | #smoking, #tobacco, #cigs, #cigarettes | health |
| There is no safe level of exposure to secondhand smoke. It causes 40,000+ US deaths per year. | #smoking, #tobacco, #cigs, #cigarettes | health |
| On average, every cig reduces your life by 11 minutes. Even occasional #smoking can hurt you. |  | health |
| Even occasional #smoking can hurt you. On average, every cig reduces your life by 11 minutes. |  | health |
| #Smoking half a pack per day costs about $1000/year. Smoking can do serious damage to your wallet. |  | money |
| #Smoking can do serious damage to your wallet. Smoking half a pack per day costs about $1000/year. |  | money |
| Formaldehyde is used to embalm dead bodies. It is also a cancer-causer in #cigarette smoke. |  | health |
| Formaldehyde is a cancer-causer in #cigarette smoke. It is also used to embalm dead bodies. |  | health |
| Polonium-210 is a chemical in nuclear reactors. It’s also found in #cigarette smoke. |  | health |
| Polonium-210 is a chemical in #cigarette smoke. It's also found in nuclear reactors. |  | health |
| Hydrogen cyanide is found in rat poison. It’s also in #cigarette smoke. |  | health |
| Hydrogen cyanide is found in #cigarette smoke. It's also in rat poison. |  | health |
| If nobody smoked, about 30% of cancer deaths in the US could be prevented. | #smoking, #tobacco, #cigs, #cigarettes | health |
| If nobody smoked, about 160,000 cancer deaths in the US could be prevented. | #smoking, #tobacco, #cigs, #cigarettes | health |
| You can never buy back time, so keep it #tobaccofree. #Smoking can shorten your life by more than 12%. |  | health |
| You can never buy back time, so keep it #tobaccofree. #Smoking can shorten your life by 10 years. |  | health |
| In the US, about 20% of all deaths are caused by a #smoking-related disease every year. |  | health |
| In the US, 480,000 deaths are caused by a #smoking-related disease every year. |  | health |
| #Tobacco use causes ~20% of all US deaths-more than AIDS, alcohol, car accidents, homicides & illegal drugs combined |  | health |
| #Tobacco use causes 1300 US deaths daily-more than AIDS, alcohol, car accidents, homicides & illegal drugs combined |  | health |
| About 30% of cancer deaths in the US are linked to #smoking. |  | health |
| Over 160,000 cancer deaths in the US every year are linked to #smoking. |  | health |
| About 40% of non-smokers in this country are exposed to toxic secondhand smoke. | #smoking, #tobacco, #cigs, #cigarettes | health |
| Over 100 million non-smokers in this country are exposed to toxic secondhand smoke. | #smoking, #tobacco, #cigs, #cigarettes | health |
| About 50% of 3-11 year-olds are exposed to secondhand smoke. There is no safe level of exposure. | #smoking, #tobacco, #cigs, #cigarettes | health |
| Over 15 million 3-11 year-olds are exposed to secondhand smoke. There is no safe level of exposure. | #smoking, #tobacco, #cigs, #cigarettes | health |
| Secondhand smoke causes 40000+ US deaths per year, and 75% are from coronary heart disease. | #smoking, #tobacco, #cigs, #cigarettes | health |
| Secondhand smoke causes 40000+ US deaths per year, including 30000+ coronary heart disease deaths. | #smoking, #tobacco, #cigs, #cigarettes | health |
| How does #smoking take a decade of life away? Smokers die about 12% earlier than non-smokers. |  | health |
| How does #smoking take a decade of life away? Smokers die about 10 years younger than non-smokers. |  | health |
| Even occasional #smoking can hurt you. Every #cigarette you smoke reduces your life by almost 20% of an hour. |  | health |
| Even occasional #smoking can hurt you. Every #cigarette you smoke reduces your life by 11 minutes. |  | health |
| If you smoke, you may be cuttin’ your time with the fam short. #Smoking can shorten your life by over 12%. |  | health + family |
| #Smoking can shorten your life by over 12%. If you smoke, you may be cuttin’ your time with the fam short. |  | health + family |
| If you smoke, you may be cuttin’ your time with the fam short. #Smoking can shorten your life by 10 years. |  | health + family |
| #Smoking can shorten your life by 10 years. If you smoke, you may be cuttin’ your time with the fam short. |  | health + family |
| Forget death, chase life. About 20% of all US deaths are caused by a #smoking-related disease. |  | health |
| About 20% of all US deaths are caused by a #smoking-related disease. Forget death, chase life. |  | health |
| Forget death, chase life. 480,000 US deaths are caused by a #smoking-related disease every year. |  | health |
| 480,000 US deaths are caused by a #smoking-related disease every year. Forget death, chase life. |  | health |
| Smoking can cause cancer almost anywhere in the body. About 30% of US cancer deaths are linked to #smoking. |  | health |
| About 30% of US cancer deaths are linked to #smoking. Smoking can cause cancer almost anywhere in the body. |  | health |
| Smoking can cause cancer almost anywhere in the body. 160,000+ US cancer deaths every year are linked to #smoking. |  | health |
| 160,000+ US cancer deaths every year are linked to #smoking. Smoking can cause cancer almost anywhere in the body. |  | health |
| Protect your loved ones by living #tobaccofree. About 40% of US non-smokers are exposed to toxic secondhand smoke. |  | health + family |
| About 40% of US non-smokers are exposed to toxic secondhand smoke. Protect your loved ones by living #tobaccofree. |  | health + family |
| Protect your loved ones by living #tobaccofree. 100 million+ US non-smokers are exposed to toxic secondhand smoke. |  | health + family |
| 100 million+ US non-smokers are exposed to toxic secondhand smoke. Protect your loved ones by living #tobaccofree. |  | health + family |
| Look out for the lil ones by keeping it #tobaccofree. About 50% of 3-11 year-olds are exposed to secondhand smoke. |  | health + family |
| About 50% of 3-11 year-olds are exposed to secondhand smoke. Look out for the lil ones by keeping it #tobaccofree. |  | health + family |
| Look out for the lil ones by keeping it #tobaccofree. Over 15 million 3-11 year-olds are exposed to secondhand smoke |  | health + family |
| Over 15 million 3-11 year-olds are exposed to secondhand smoke. Look out for the lil ones by keeping it #tobaccofree |  | health + family |
| #Tobacco is a major preventable cause of death. If nobody smoked, ~40% of US cancer deaths could be prevented. |  | health |
| If nobody used tobacco, ~40% of US cancer deaths could be prevented. #Tobacco is a major preventable cause of death |  | health |
| #Tobacco is a major preventable cause of death. If nobody used tobacco, ~230,000 US cancer deaths could be prevented |  | health |
| If nobody used tobacco, ~230,000 US cancer deaths could be prevented. #Tobacco is a major preventable cause of death |  | health |
| When someone dies from #tobacco use, we lose them too soon. Smokers die about 12% earlier than non-smokers. |  | health + community |
| Smokers die about 12% earlier than non-smokers. When someone dies from #tobacco use, we lose them too soon. |  | health + community |
| When someone dies from #tobacco use, we lose them too soon. Smokers die about 10 years younger than non-smokers. |  | health + community |
| Smokers die about 10 years younger than non-smokers. When someone dies from #tobacco use, we lose them too soon. |  | health + community |
| Even occasional #smoking can hurt you. If nobody smoked, about 30% of US cancer deaths could be prevented. |  | health |
| If nobody smoked, about 30% of US cancer deaths could be prevented. Even occasional #smoking can hurt you. |  | health |
| Even occasional #smoking can hurt you. If nobody smoked, about 160,000 US cancer deaths could be prevented. |  | health |
| If nobody smoked, about 160,000 US cancer deaths could be prevented. Even occasional #smoking can hurt you. |  | health |
