# Supplementary material for: General Audience Engagement With Antismoking Public Health Messages Across Multiple Social Media Sites: Comparative Analysis
Source: JMIR Public Health Surveill. 2021 Feb 19;7(2):e24429. doi: 10.2196/24429 (PMC7935649; doi:10.2196/24429)
Supplement: Multimedia Appendix 2 [file publichealth_v7i2e24429_app2.docx]

**Multimedia Appendix 2.  Targeting criteria for paid messages (advertisements) on Twitter, Facebook, and Instagram.**

|  | **Twitter** | **Facebook & Instagram** |
| --- | --- | --- |
| **Campaign type** | Website visits or conversion | Traffic (Website visits) |
| **Ad type** | Promoted tweet | Boosted post (Facebook)  Promoted Ad (Instagram) |
| **Campaign (message) duration** | 2 days | 2 days |
| **Location** | United States | United States |
| **Gender** | All (Any) | All (Any) |
| **Age** | N/A | 13-65+ |
| **Language** | English | English (US) |
| **Keywords** | 1. CDC Tobacco Free 2. Cigarette 3. Electronic cigarette 4. Food and Drug Administration 5. Health and Well Being 6. Smoking 7. Smoking cessation 8. Stop Smoking Now 9. Tobacco 10. Tobacco smoking people | Not available on Facebook or Instagram |
| **Interests** | None | 1. CDC Tobacco Free 2. Cigarette 3. Electronic cigarette 4. Food and Drug Administration 5. Health and Well Being 6. Smoking 7. Smoking cessation 8. Stop Smoking Now 9. Tobacco 10. Tobacco smoking people |
