# Supplementary material for: General Audience Engagement With Antismoking Public Health Messages Across Multiple Social Media Sites: Comparative Analysis
Source: JMIR Public Health Surveill. 2021 Feb 19;7(2):e24429. doi: 10.2196/24429 (PMC7935649; doi:10.2196/24429)
Supplement: Multimedia Appendix 4 [file publichealth_v7i2e24429_app4.pdf]

Table 4. Effects of message type (paid ad vs. organic messages) on wCTR for the analysis of 1275 antismoking health messages that were posted across 3 social media platforms (Twitter, Facebook, and Instagram) between April 19 and July 12, 2017.

Variable	N	wCTR (N=700)	Comparison	*P*[a]
**Ad type**	700		Organic vs. Paid	P<.001
**Organic**	78	0.0384 (0.0006, 0.0762)		
**Paid**	622	0.1719 (0.1531, 0.1906)		

[a]*P*-values were obtained using multilevel mixed-effects negative binomial regression, followed by calculation of marginal means.
